# Effect of Incorporating Date Seeds Microparticles on Compressive Strength and Microhardness of Conventional Glass Ionomer (an *In Vitro*Study)

**DOI:** 10.4317/jced.61603

**Published:** 2024-07-01

**Authors:** Abeer G. Abdulkhaliq, Bashaer A. Najim

**Affiliations:** 1 Aesthetics and Restorative Department, College of Dentistry, University of Baghdad, Baghdad-Iraq

## Abstract

**Background:**

This study aimed to evaluate the effect of incorporating date seeds (DS) microparticles on the compressive strength and microhardness of conventional glass ionomer cement properties following aging in artificial saliva.

**Material and Methods:**

Date seeds powder was prepared and added to the conventional glass ionomer cement at concentrations of 3% and 5% by weight. To prepare the samples, a silicon mold was utilized, with dimensions of 6 mm in height and 4 mm in diameter. These samples were then divided into three main groups: group I; unmodified GICs serving as the control, group II; GICs with a 3% weight of DS, and group III; GICs with a 5% weight of DS. The compressive strength and microhardness of the samples were subsequently measured and compared across the three groups, after being stored in artificial saliva for two different time intervals: one day and 30 days. Fourier transform infrared (FTIR) analysis was conducted to determine the nature of the DS and the GIC-DS composite. At the same time, a scanning electron microscope (SEM) was employed to investigate the surface characteristics of the samples.

**Results:**

The measurement values after 24 hours showed that the DS addition had significantly increased the compressive strength but had no effect on the microhardness. However, after aging there was a significant increase in the microhardness and a significant decrease in the compressive strength of the DS groups compared to the control group.

**Conclusions:**

The addition of date seeds powder showed an enhancing effect on the microhardness over time but adversely affected the compressive strength of the material.

** Key words:**Artificial saliva, natural resources, waste materials, dental restoration, mechanical properties.

## Introduction

Glass-ionomer cement (GIC) is a restorative dental material with a variety of applications. It was first used by Wilson *et al*. at the Government Chemist Laboratory in early 1970 after their studies on dental silicate cements and zinc polycarboxylate cements (1,2). Glass ionomer cement is an acid-base cement whose fundamental components are inorganic fluoroaluminosilicate glass powder and weak, water-soluble polyacrylic acid (PAA) (3,4). A gel phase is reached when the acid combines with the glass particles. Higher viscosity paste is created, and it progressively hardens over a few minutes. Ultimately, this process produces a rigid composite of the gel matrix reinforced by glass particles. Aluminosilicate glass also contains calcium in its basic form, phosphate may be included to the mixture to improve the glass network’s development through its reaction with aluminum. Furthermore, fluoride is added to the mixture in order to take the advantage of the set cement’s fluoride release and lower the melting temperature of the glass mix. The result is glass that is fluoroaluminosilicate (5-7). The favorable characteristics of GICs include biocompatibility (5), long-term release of fluoride serving as an anti-cariogenic agent, modulus of elasticity similar to dentin, suiTable coefficient of linear thermal expansion and contraction (8), and direct bonding to the tooth structure (1,9,10). It is utilized in many different applications, such as the restoration of primary teeth, as a filling material for dental cervical lesions, core construction, and a traumatic restorative treatment (ART) (11,12). One of the primary drawbacks of this kind of material is that its poor mechanical properties restrict its application as a posterior restoration (13). Therefore, more research is being done to enhance the material’s mechanical properties. The cement powder and liquid have been modified in various ways by adding a variety of materials, such as bioactive apatite (14), zinc oxide (15) and basalt fibers (16). Moreover, because of growing awareness of the negative environmental effects of synthetic materials and growing demand for sustainable and renewable alternatives, researchers are looking at the reinforcing effects of organic elements (17,18). Researchers have been interested in date seeds since they are a naturally occurring substance that can be found for almost no cost, are simple to manufacture, are biocompatible, and are environmentally friendly (19). The date seed is a hard-coated, often rectangular, ventrally grooved seed with a small embryo. Depending on maturity, variety, and grade, it can weigh anywhere from 0.5 to 4 g, or 6 to 20% of the weight of the fruit (20). In a study by Nwogu *et al*. (21), it had been found that date seed reinforced composites provide excellent mechanical properties at a lower cost than glass fiber composites. To create improved polymer nanocomposites, date seed organic nanoparticles were used as a filler to polyethylene terephthalate (PET) (22). It was discovered that adding a date seeds powder reinforcement enhanced and optimized the composite’s main mechanical properties, including hardness, compressive strength, and wear resistance (21).

The objective of this study was to evaluate the mechanical properties (compressive strength and microhardness) of conventional glass ionomer cement after the incorporation of date seed powder at a concentration of 3% and 5% by weight. The tested hypothesis was that the incorporation of date seeds microparticles with different proportions into conventional glass ionomer would have no significant effect on the tested mechanical properties.

## Material and Methods

-Sample preparation 

A commercially available conventional Fluoroaluminosilicate glass powder consisting of SiO2, Al2O3, CaF2 and poly acrylate solution (Medifil, PROMEDICA Dental Material GmbH, Germany) was used in this study. Waste of palm date was also used (Iraqi Bareem Dates). The date seeds were collected and soaked in water, the date flesh adhering to the seeds was removed through a process of washing. Subsequently, the seeds were allowed to dry naturally for 24 hour. To further ensure complete dryness, the seeds were placed in an air-oven at a temperature of 60°C. Following this, the seeds were subjected to crushing and milling for duration of 5 hours. This was achieved using a high-speed multi-functional crusher with a rotating speed of 22000r/min. The material was passed through a filtering screen until the target micro scale particle size (less than 500 μm) was reached. The powder was then kept in the freezer at -20° C. until used (23).

The experimental GIC was prepared by adding DS at weight fractions of 3%, and 5% of the total powder amount. An electronic balance with three digits accuracy was used to prepare the mixed powders. The fluoro-aluminosilicate glass powder and date seed microparticles powder were then manually mixed with a mortar and pestle for 10 min to obtain as homogenous distribution. Afterward, the prepared powder was mixed with the poly acrylic solution at 2/1 powder / liquid ratio according to the manufacturer’s instructions.

The mixed cement was transferred into a silicon mold conforming to the dimensions specified in the ISO 9917-1 standard(24) (6 mm in height and 4 mm in diameter). Subsequently, the samples were placed in an incubator at 37 °C for a period of 1 hour to allow for material hardening. The samples were removed from the mold and stored in a tube containing 10 mL of artificial saliva (25) at room conditions until further investigation (26,27).

There were three experimental groups as follow:

Group I: Unmodified conventional glass ionomer (control group).

Group II: Glass ionomer cements modified with date seed microparticle 3% by weight of the total powder.

Group III: Glass ionomer cements modified with date seed microparticle 5% by weight of the total powder.

These groups were further divided in two subgroups according to storage time; 1 day and 30 day of aging in artificial saliva.

-Mechanical testing 

Compressive strength

**Figure 1:**
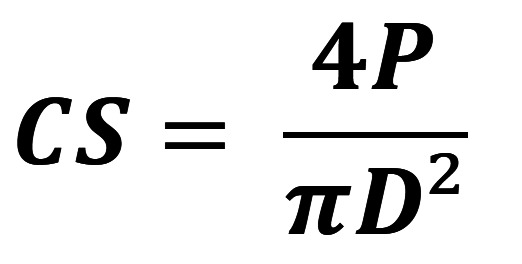
Formula.

For the purpose of compressive strength analysis, a total of 12 specimens were carefully prepared, with an even distribution of 6 samples per time point. The specimens were placed in a vertical orientation, with the force being applied along their longitudinal axis. Subsequently, the specimens were subjected to compression within a universal testing machine, utilizing a standardized a crosshead speed of 0.5 mm/min, until the point of fracture was reached. The compressive strength was subsequently calculated employing the following equation: (Fig. 1).

Where CS is the compressive strength (MPa), *P* is the maximum applied load (N) and D is the diameter of the sample (mm).

-Vickers microhardness

For each main group, a total of 12 specimens were prepared for the Vickers microhardness examination, with an equal distribution of 6 samples per time point. Employing a highly precise micro-indentation device, namely the Micro Hardness Tester, HVS-1000, courtesy of LARYEE TECHNOLOGY CO., LTD, the Vickers hardness numbers (VHN) were determined. This process involved the application of a 50 g load for duration of 10 seconds. The indenter was meticulously placed at the center of the specimen, and upon removal of the aforementioned load, a thorough examination was conducted using a microscope for measuring the lengths of the two diagonals left behind by the indenter on the material’s surface, the average diagonal was recorded. For each individual sample, the average of three microhardness readings was obtained.

-Scanning electron microscope (SEM)

Scanning electron microscope (SEM) NOVA (Model: NANOSEM 230, FEI Company, Hillsboro, OR, USA) was used to progressively characterize the fracture surfaces of the coated fractured specimens of glass-ionomer cement, over a range of magnifications. Fractured half of one specimen from each sample was glued on a specimen holder and vacuum sputter coated with a thin layer of gold for conductivity.

-FTIR 

An attenuated total reflection-Fourier transform IR (Spectrum Two N, PerkinElmer, Inc. USA) was employed to identify the component of the powder of raw date seeds and to evaluate the reactions of experimental GICs in comparison to control group at room temperature (20°C). An aluminum holder was used to keep the powdered substance in place while it was tested. For the acquisition of the FTIR spectra, a liquid N2-cooled MCT (HgCdTe) detector was utilized. For these scans, the scan depths ranged anywhere from 400 cm-1 to 4000 cm‾¹. Each specimen’s spectrum was compared to the diffuse reflectance spectrum of pure KBr, which served as the background spectrum for the experiment.

-Statistical analysis

Data were submitted to the Shapiro-Wilk normality test and analyzed parametrically as the data followed a normal distribution. One-way analysis of variance (ANOVA) and tukey post hoc tests were used to evaluate significances (alpha level = 0.05) in mean values amongst the tested groups at each time interval. Moreover, independent t-tests (*p* < 0.05) were also used to evaluate the effects of different storage duration on the mechanical properties per each group. All analyses were conducted using SPSS statistical package (version 24; SPSS®Inc., IBM®, Chicago, IL, USA).

## Results

-Compressive strength

**Figure 2:**
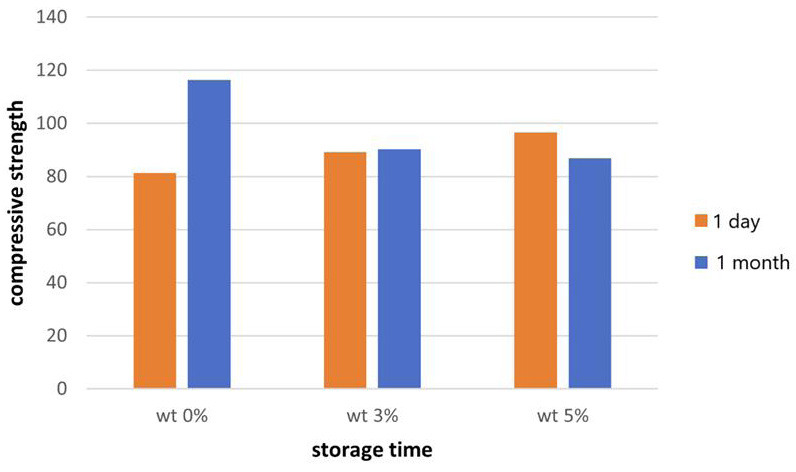
Bar chart showing the mean values of compressive strength of different groups.

The mean values of the compressive strength of different groups measured after 1 day and 30 days storage in artificial saliva are shown in Fig. 2.

The measured values after one day showed an increase in the compressive strength in the groups of DS compared to the control group and that increase was statistically significant (*p*<0.00). Also, increasing the weight percentage of DS led to significantly higher compressive strength (*p*<0.00) ( Table 1).

After one month storage in artificial saliva, group I had the highest compressive strength mean value, which was statistically significantly higher (*p*<0.05) in comparison to their 1 day storage group, group II and group III, while the differences between group II and group III were not statistically significant (*p*>0.05). There was a statistically significant effect of one month aging in increasing the compressive strength in the group I, while for group III the compressive strength decrease significantly with no effect found in group II ( Table 1).

-Microhardness

**Figure 3:**
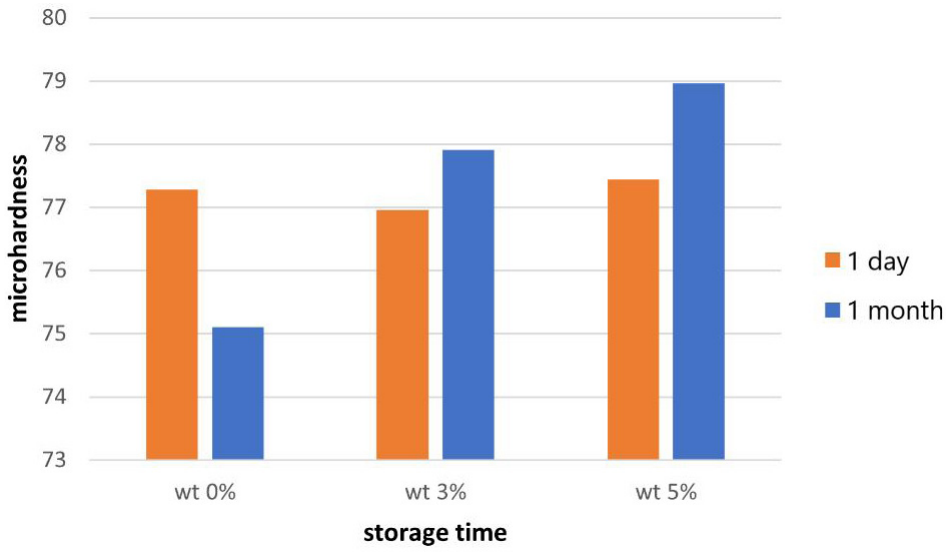
Bar chart showing the microhardness means values after 1 day and 1 month duration.

The microhardness mean values for the three group after storage for 1 day, 30 days are shown in the Fig. 3.

After 24 hours, the highest mean value of microhardness was found in group III (5%). However, the difference between the groups was statistically insignificant (*p*>0.05) ( Table 2). Measurements after 1 month showed that the highest recorded mean value was also in group III with statistically significant difference between the groups. Further data analysis with post hoc tukey test showed that the microhardenss increased significantly after increasing the weight of the DS from 3% to 5%. The aging didn’t have a significant effect in group II. Meanwhile, the aging cause significant differences in the microhardness of the control group and group III.

-Scanning electron microscopy 

**Figure 4:**
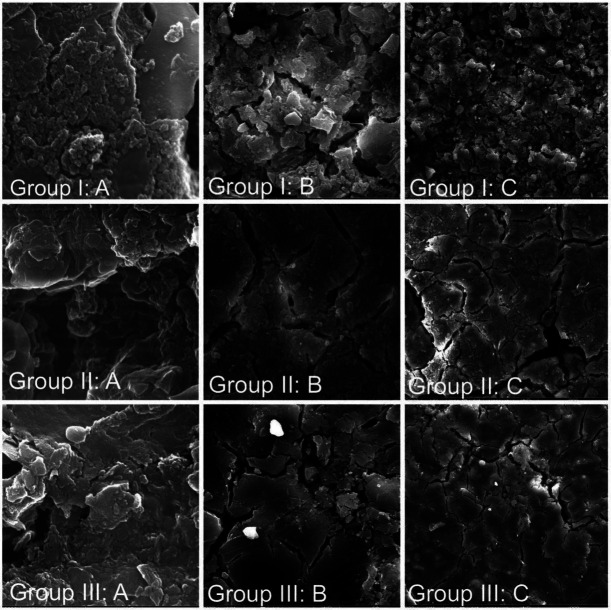
Scanning electron micrograph of the fractured surfaces of the compressive test samples after 1 month immersion in artificial saliva, at (A) x50000, (B) x2000, (C) x500.

The microphotograph obtained from the scanning electron microscope exhibits non-reacted glass cores that are surrounded by an intermediate layer of siliceous hydrogel. These glass cores are embedded within a cross-linked polysalt gel cement matrix (4).This particular microstructure can be observed in all of the conventional GICs (26,27). Scanning electron micrographs (Fig. 4) demonstrate regions of the fractured surfaces for experimental and control GICs, using a different magnification, after 30 day of aging in artificial saliva. The micrographs for all tested groups showed numerous profound cracks that might be originated as a result of dehydration during the SEM test preparation or might be developed during the compressive testing. The surface of the control group samples showed more glass particles of different sizes bonded to the polymer matrix, causing the surface to appear rougher in comparison to the other experimental groups which appear to be smoother with less exposed glass particles. On the other hand, the micrograph of modified GICs (group II and group III) showed more voids that were generally appear greater in depth and size than control. Fracture surfaces of group II sample contain more glass particles than group III, whereas more cracks and void were observed in lower-magnification photomicrographs of group II fracture surfaces. Moreover, all experimental samples, exhibited signs of minimal mineral depositions on their surface, which were dissimilar from the particles within the cement most clearly evident in group III photomicrograph (Fig. 4; Group III).

-Fourier-transform infrared (FTIR) spectroscopy 

FTIR of the raw date seeds powder showed a wide peak at 3375.25 cm‾¹ may be from the hydroxyl group (O-H) in water molecules, as date seeds contain hemicellulose and cellulose in their fiber. Alkanes stretching peaks indicate carbon and hydrogen. Absorption band at 1746.77 cm‾¹ correspond to carbonyl group. Adsorption peak at 1647.97 cm‾¹ indicates presence of alkenes or aromatic compounds.

**Figure 5:**
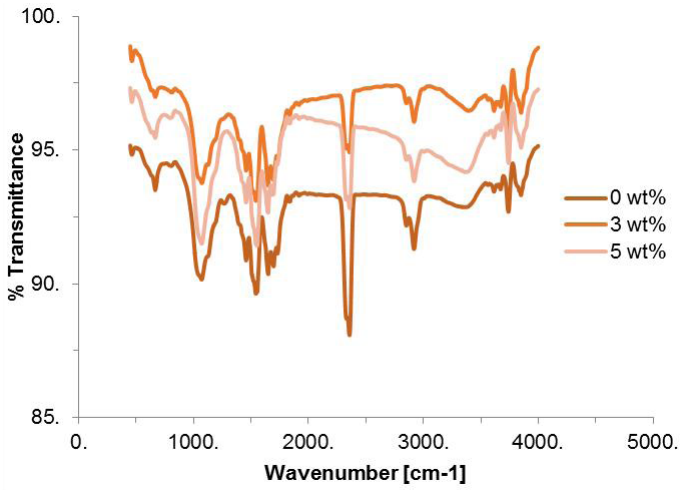
The FTIR spectroscopy for the control and the experimental groups.

The FTIR showed that the absorption peaks for the different groups were almost similar for all tested percentage of date-seed modified and unmodified glass ionomer but the peaks was more prominent for the control group (Fig. 5). The stretching of the (C-H) carboxylic acid aliphatic group is attributed to the vibrational modes observed at wave numbers 2919-2365 cm‾¹. Additionally, the stretching of siloxane at 1082 cm‾¹ and the bending of Si-OH at 954 cm‾¹are also observed. A broad peak is associated with the alpha helix amide bond. The peaks at approximately 1555 and 1631 cm‾¹represent polyacrylate salts with mono/divalent counter ions, while the peaks at 1544 cm‾¹ indicate the presence of (C=C) Alkene. The presence of carbohydrates is indicated by a peak at 1064 cm‾¹. Different wave numbers are observed for the stretching vibrations of halogen groups. The absorption peaks at 460 cm‾¹, 750 cm‾¹, and 1028 cm‾¹ correspond to Si-O vibrations. The N-H vibration is represented by the peak at 1600 cm‾¹. The O-H stretching originating from water or the -Si-OH is assigned to the peak at 3441 cm‾¹.

## Discussion

Improving the mechanical properties of GIC by incorporating date seeds powder was the subject of evaluation of this study. Date seeds is a natural organic material that is available a waste product, and has been found to be a potential reinforcing material (19,21,22,28-30). Fourier-transform infrared (FTIR) spectroscopy of raw seed powder, indicated the presence of various constituents, including water, carbohydrates, proteins, and fatty acids. FTIR spectroscopy has confirmed the existence of alkanes group, carbon, hydrogen, and carbonyl group (C=O) in the date seeds. Additionally, the FTIR analysis revealed the presence of the C=C double bond stretching, indicating the presence of alkenes or aromatic group compounds. The FTIR analysis also showed the stretching of both the C=O group and the N-H bending vibrations of proteins. The amino structure in the date seeds is capable of forming hydrogen bonds with -COOH along the polycarboxylic acid chain and with the silica gel on the unreacted particle surface (31). Furthermore, the amino structure could also chelate with ions such as Al+3 and Ca+2, thereby participating in the formation of an ions crosslinked network structure (32,33) All of these factors could contribute to improve the adhesion between the date seeds and the GIC.

Various glass powder and polyacid liquid formulations have been developed over time to enhance the mechanical properties of conventional GIC (34-39). Moreover, Kheur *et al*. reported a significant enhancement in flexural strength and shear bond of conventional GIC by adding 6% weight percent Hydroxyl Apatite particles ranging from 80 to 150 nm at a liquid to powder ratio of 3:1 (40). In contrast, other studies have demonstrated that the addition of bioactive glass particles causes a noticeable decline in the mechanical strength of the cements, which was attributed to the partial substitution of conventional ions in the overall powder composition, resulting in a decrease in the proportion of Al3+ in the glass mixture. Consequently, weaker bonds are formed between the PAA and other released ions, such as Ca2+ (41-43). On the other hand, Neven and Aref, in their study, discovered that sesame oil demonstrated the capacity to modify the physical characteristics of traditional Glass Ionomer Cement (GIC) post its integration into the cement’s liquid component at ratios of 3 and 5 (v/v%). Both the compressive and diametral tensile strengths of the sesame oil-modified cement at 3 and 5 (v/v%) exhibited a significant enhancement. This was ascribed to sesame oil’s capability to enhance the level of cross-linking and interlocking within the cement structure (44).

Among the properties of the GIC, compressive strength, roughness, and microhardness are particularly important. The compressive strength of the material determines its ability to withstand specific stresses (45), while the microhardness of the material measures its resistance to permanent deformation. Therefore, improvements in these properties can enhance the performance of restorative materials during clinical applications (46). It is important to note that GIC materials are sensitive to initial desiccation and moisture (47). As GIC serves in a moist oral environment, it is not only affected by the water content within the material itself but also eroded by water absorbed from the external environment. Additionally, the carboxylate structure in GIC is in a constant dynamic process of formation and hydrolysis, leading to changes in its mechanical properties. Therefore, studying the aging resistance properties of GIC is of particular importance (48).

The increase in compressive strength of conventional glass ionomers during the initial period of a few weeks was established decades ago (4,49,50). After the initial setting of chemically set glass ionomer cements (GICs), the setting reaction typically continues for a few days during the maturation process. This period is characterized by the initial accumulation of calcium polyalkenoate, followed by the formation of aluminum polyalkenoate, which ultimately influences the final mechanical properties of the material (51,52). According to Williams and Billington, conventional GICs based on polyacrylic acid demonstrate a constant or even a slight increase in their compressive strength over the time span of 24 hours to 4 months (53). Similarly, Nicholson (4) pointed out that air voids in glass ionomers likely contain gel structures, which gradually mineralize from the edges inward as relevant ions diffuse into them. This gradual mineralization process could potentially enhance the strength of the cement, thereby serving as one of the mechanisms responsible for the increase in compressive strength during maturation. These findings may provide a reasonable explanation for the significant increase in compressive strength observed in group I after 30 days of aging.

However, a study by Jowkar *et al*. 2019 found that adding silver nanoparticle to conventional glass ionomer cement in 0.1% and 0.2% (w/w) improved the compressive strength, surface microhardness, and micro shear strength to dentin compared to the unmodified GIC, suggesting that the small sizes of the silver nanoparticles incorporated and the high density of interfaces of nanomaterial and the tendency of nanoparticles to resist the compression forces may justify the improvement of the flexural and compressive strengths of the modified GIC. This was agreement with the results of 1 day aging in our study, as the aging process was not clear in their study, suggesting that their results were immediate or after short storage period (54).

According to this study, group II and III’s compressive strength significantly enhanced after a 24-hour period when compared to the GIC control group. Moreover, group III, which had the largest date seeds powder weight percentage, produced better outcomes. The incorporation of date seeds powder microparticles may improve the GIC material’s properties by filling the gaps between the glass particles and offering more bonding sites for polyacrylic polymers during initial setting reaction (50). Increasing the weight of date seeds microparticles led to more voids being occupied and hence higher strength. The date seeds particles likely functioned as fillers that were not firmly adhered to the GIC matrix, making the material more susceptible to water absorption, deteriorations, and loss of strength during aging in artificial saliva. Consequently, after one month, the compressive strength decreased significantly compared to the GIC control group (48,55).

Although water is essential for the setting reaction of GIC, it can also cause degradation of the carboxylate structure (56,57) through either substituting the COO-1 ligand with OH-1, thereby disrupting the ionic bond between the metal ions and the polycarboxylate acid chains, or it can replace the COO-1 ligand with H2O, resulting in a reduction in the strength of the ionic bond (58). This phenomenon is further exaggerated in the oral environment due to the presence of aggressive compounds in saliva (50). The incorporation of particles may also interfered with the reactions occurring during the maturation phase, thereby reducing the formation of polysalt (51). The justification for this claim is further supported by SEM photomicrographs, which displayed the presence of surface microporosities and inadequate bonding between the particles of date seed and the matrix (Fig. 3). According to Xie *et al*. (59), the SEM photomicrograph which revealed denser surface textures, fewer and smaller voids, and smaller particles, which appear to have resulted in a higher compressive strength for group I. Conversely, the SEM photomicrographs of the group II and III showed less dense surface textures, and more voids, which may have caused opposite trends in the compressive strength values.

The observed significant decrease in compressive strength after aging could also be attributed to the alterations in the composition and P/L ratios of GIC, as it’s directly influence their properties (60). It is worth noting that the mechanical properties of the set cement are determined by the powder-to-liquid ratio. An increase in powder content enhances the consistency, strength, and speed of the setting reaction (61). However, in the case of the modified GIC with date seeds, the weight of the additive replaced part of the amount of GIC powder in the base material, while the GIC solution remains constant. Consequently, the lower powder-to-liquid ratio of the date seed modified GIC may lead to an increase in solubility (62). the clustered water at the interface has the potential to hydrolyze any bonds that could form between the date seed powder and the matrix (63).

The findings were consistent with previous investigations; the examination of the incorporation of zirconia fillers into GICs revealed an increase in compressive strength after a duration of 24 hours, however, subsequent aging resulted in a decrease in compressive strength (64). Another investigation conducted by Elshenawy *et al*. explored the impact of incorporating quaternized chitosan-coated mesoporous silica nanoparticles (HTCC@MSNs) into conventional GIC, which led to a reduction in strength after aging (65). Wang and Darvell (55) concluded that storage in a wet environment adversely affects ceramic-reinforced glass ionomers, suggesting potential long-term deterioration during clinical use. An research conducted by Al-Taee *et al*., which involved the inclusion of finely sized reactive hydroxyapatite and fluoroapatite particles (<6%) within the glass powder, did not demonstrate any advantageous effect in terms of mechanical strength, resulting in a cement with inferior mechanical properties (6).

Microhardness is a surface property that has an impact on the performance of GICs (66). The microhardness values exhibited a slight decrease in group II but an increase in group III after 24 hours. However, these changes were not statistically significant. After one month of storage in artificial saliva, the microhardness significantly increased in group II and III, which aligns with the findings of Elkhouly *et al*. (22) and Al-Tae’e *et al*. (6) Previous studies have shown that the variations in microhardness of GICs depend on the maturation stage, setting reaction, and interactions with the storage medium (6,67,68). However, there is still ongoing debate regarding the long-term effect of storage on hardness of GICs. While conventional GICs stored in water tend to increase in hardness (69-73), there are reports of inverse relationships for some new compositions (26,66,69).

It can be proven that the significant decrease in surface hardness of the control group after the aging process can be ascribed to an extended secondary setting reaction that takes place during the maturation phase. This specific reaction entails the deterioration of glass particles caused by the leaching of siliceous species, leading to the formation of a silica gel framework. This finding aligns with recent studies that have discussed the mobility of proton (74) and aqueous polyacrylic acid (75) within glass ionomer cements (GICs).

An increase in the microhardness of the modified glass ionomer cements (GICs) was observed compared to the control GICs after 30 days of storage in artificial saliva. This increase in microhardness can be attributed to the debilitation caused by the erosion and plasticizing impact of water. The influence of water might have induced an increase in the diffusion of ions from the date seeds modified GICs into the saliva (76), as the incorporation of particles may have interfered with the reactions during the maturation phase, leading to more ions being available. As a result, the increase in hardness of the modified GICs is likely due to the diffusions of these ions, such as calcium and phosphate, back into the cement structure. This can be further explained by the connectivity of aluminum and phosphorus atoms in the hydrogel matrix. Phosphate ions from saliva may connect with aluminum in certain surface regions of the matrix. Calcium ions with a coordination number of six may also diffuse into the matrix and form ligands (77,78). A research by Okada *et al*. have shown that these connections occur in the subsurface region of the GICs, resulting in a thin layer on the cement’s surface (77). This thin layer has a different chemical composition and may contain calcium phosphate, aluminum phosphate, and polyacrylate salts. These compounds have strong ionic bonding and low solubility in water, including saliva.

One of the limitations of the research is the use of micro size date seeds powder with low weight percentage. The date seeds powder could further be filtered from impurities to retain higher percentage of functional components. Also, extended time of aging more than one month is advisable.

## Conclusions

The incorporation of date seeds in the form of microparticles (3% and 5% by weight) into the conventional glass ionomer cement improved the early compressive strength. However, it should be noted that after a period of 30 days of exposure to artificial saliva, there is a significant decrease in the compressive strength. On the other hand, the microhardness of the experimental GICs experiences a significant increase after one month of aging, as evidenced by the presence of minimal mineral deposition observed on the scanning electron microscope (SEM) microphotograph.

## Figures and Tables

**Table 1:** Compressive strength mean MPa( ±SD) aged for 1 day,30 days and comparison of significance among groups.

Group (n=6)	1 day	30 days
	(Mean MPa) [SD]	(Mean MPa) [SD]
I control	81.20 [.81731]*^c^	116.27 [.84533]^*^^d^
II (3% DS)	89.26 [.84301]^b^	90.27 [2.83328]^e^
III (5% DS)	96.63 [.64395]^a^	86.89 [3.00019]^*^^e^

Different letters indicate a significant difference amongst the groups (ANOVA, tukey test, an alpha level of 0.05) *indicate significant effect of aging for the same group (independent t-test).

**Table 2:** Microhardness shown as mean MPa (± SD) aged for 1 day, 30 days.

Group n=6	Mean (±SD)	Mean (±SD)
	1 day	30 day
I control	77.36[.35024]^a^	75.10[2.47053]^*^^c^
II (3% DS)	77.01[.61779]^a^	77.90[1.10812]^b^
III (5% DS)	77.50[.43818]^a^	78.97[.51315]^*^^b^

Similar letters in columns indicate no significant differences among groups (ANOVA, tukey test post-hoc tests, alpha level 0.05). *indicate significant effect of aging for the same group (independent sample t-test)

## Data Availability

The datasets used and/or analyzed during the current study are available from the corresponding author.
